# Spontaneous heterotopic mesenteric ossification around the pancreas causing duodenal stenosis: A case report with literature review

**DOI:** 10.1016/j.ijscr.2021.105702

**Published:** 2021-03-06

**Authors:** Hanlim Choi, Jae-Woon Choi, Dong Hee Ryu, Chang Gok Woo, Ki Bae Kim

**Affiliations:** a Department of Surgery, Chungbuk National University Hospital, Cheongju, Republic of Korea; b Department of Surgery, Chungbuk National University College of Medicine, Cheongju, Republic of Korea; c Department of Pathology, Chungbuk National University Hospital, Cheongju, Republic of Korea; d Department of Internal Medicine, Chungbuk National University Hospital, Cheongju, Republic of Korea

**Keywords:** Heterotopic ossification, Mesenteric ossification, Intrabdominal pseudomalignant ossification, Periampullary cancer

## Abstract

**Introduction and importance:**

Heterotopic mesenteric ossification (HMO) is a rare condition that can be hereditary or nonhereditary. It can lead to small bowel obstruction, which may require corrective surgery. Most affected patients have a history of abdominal surgery or trauma. Spontaneously occurring HMO is even rarer, with only 7 cases reported till date. There has been no previous report of spontaneous peripancreatic HMO.

**Case presentation:**

A 60-year-old man presented with complaints of recurrent nausea and vomiting for 2 months. Esophagogastroduodenoscopy revealed luminal stenosis and edematous changes involving the second and third parts of the duodenum but not its complete obstruction. Abdominopelvic computed tomography showed faintly enhanced thickening of the involved duodenal walls along with mild dilatation of the common bile duct. Considering the possibility of periampullary cancer, we performed a pylorus-preserving pancreaticoduodenectomy. Histopathological examination confirmed the diagnosis of HMO with extensive fibrosis involving the peripancreatic soft tissue.

**Clinical discussion:**

The peripancreatic HMO with severe fibrosis can occur duodenal stenosis, and it is mimicking periampullary cancer. However, the preoperative diagnosis of spontaneous HMO is difficult, and a diagnosis confirmed after surgery.

**Conclusion:**

Herein, we described our experience of managing a rare case of duodenal stenosis due to spontaneous HMO involving peripancreatic tissue.

## Highlights

• Heterotopic mesenteric ossification (HMO) is a rare condition that can lead to small bowel obstruction, which may require corrective surgery.
• Spontaneously occurring HMO can be occurred, but it is very rare.
• The peripancreatic HMO with severe fibrosis can occur duodenal stenosis, and it is mimicking periampullary cancer.
• Large heterotopic ossifications can be diagnosed by radiologic examination, but in small cases it is difficult to diagnose except for histological examination after surgery.

## 1 Introduction

Heterotopic mesenteric ossification (HMO) is defined as the development of a bony lesion within the intra-abdominal mesentery [[1], [2], [3]]. There are <40 cases of HMO reported in the literature [3]. However, a spontaneous presentation is extremely rare, and there have been no reported cases of HMO surrounding the pancreas. Here, we describe a rare case of a patient diagnosed with spontaneous HMO involving peripancreatic tissue, a presentation mimicking that of periampullary cancer. This work has been reported in line with the Surgical Case Reports guidelines [4].

## 2 Presentation of case

A 60-year-old man presented with complaints of recurrent nausea and vomiting for 2 months. No history of abdominal surgery or trauma was noted. There were no specific abnormalities in the laboratory analyses, carcinoembryonic antigen (CEA) and carbohydrate antigen 19-9 (CA19-9). Abdominal X-ray indicated a mildly distended stomach.

**Fig. 1:**
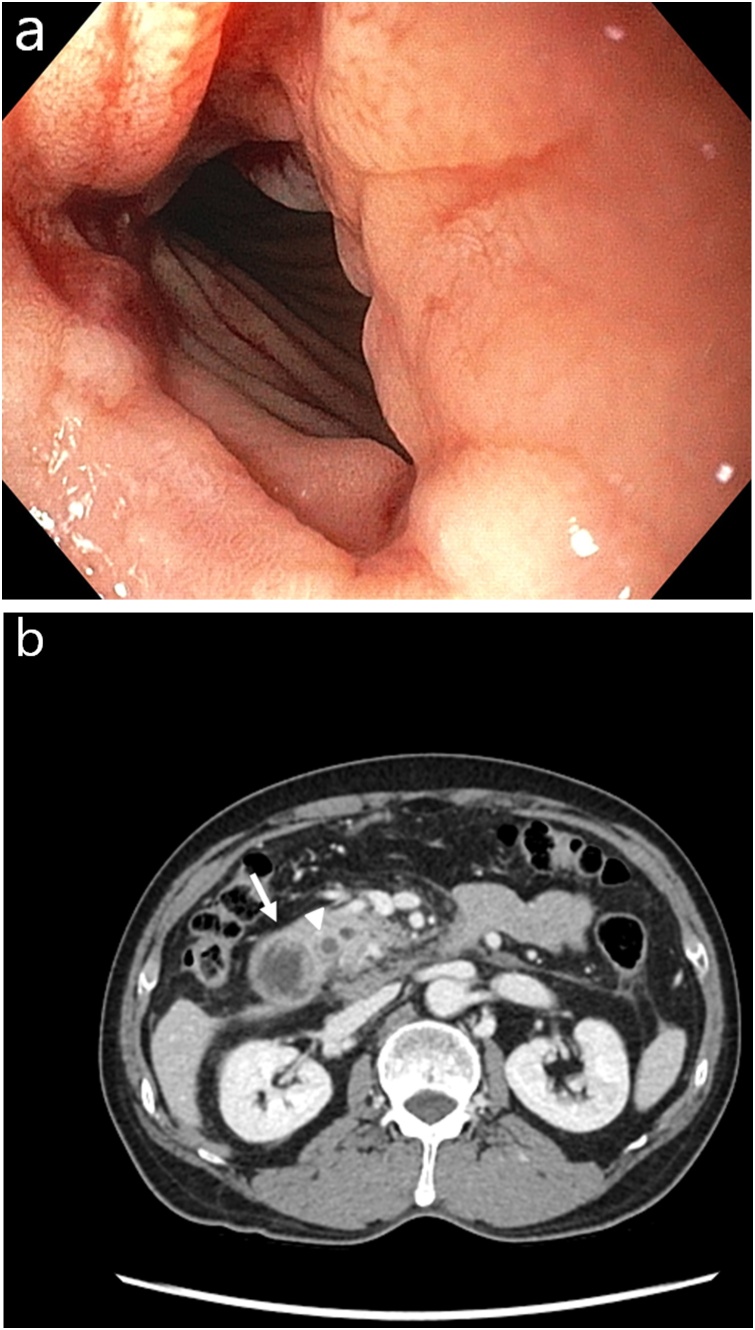
Preoperative imaging features. (A) Esophagogastroduodenoscopy image showing luminal stenosis and edematous changes affecting the second and third parts of the duodenum, though the involved lumen is not obstructed completely. (B) Computed tomography scan of the patient’s abdomen and pelvis showing faintly enhanced thickening of the involved duodenal walls (arrow) along with mild dilatation of the common bile duct (arrowhead).

We inserted a nasogastric tube and drained 50–300 ml of bile-stained gastric fluid for 3 days. After the stomach was fully decompressed, we performed an esophagogastroduodenoscopy (EGD), which showed both a large amount of bile fluid and food material in the stomach. The EGD further revealed luminal stenosis and edematous changes affecting the second and third parts of the duodenum, though the involved lumen was not obstructed completely (Fig. 1A). Computed tomography (CT) images of the patient’s abdomen and pelvis showed faintly enhanced thickening of the involved duodenal walls along with mild dilatation of the common bile duct (Fig. 1B).

Considering the possibility of cancer of either the pancreatic head or the periampullary region, we performed an exploratory laparotomy. Intraoperatively, we observed massive fibrosis and adhesions surrounding the second and third parts of the duodenum and the head of the pancreas. The entire pancreatic body was hardened, and the connective tissue around the head of the pancreas showed severe desmoplastic changes. We performed sophisticated adhesiolysis to release the duodenum along with a pylorus-preserving pancreaticoduodenectomy.

**Fig. 2:**
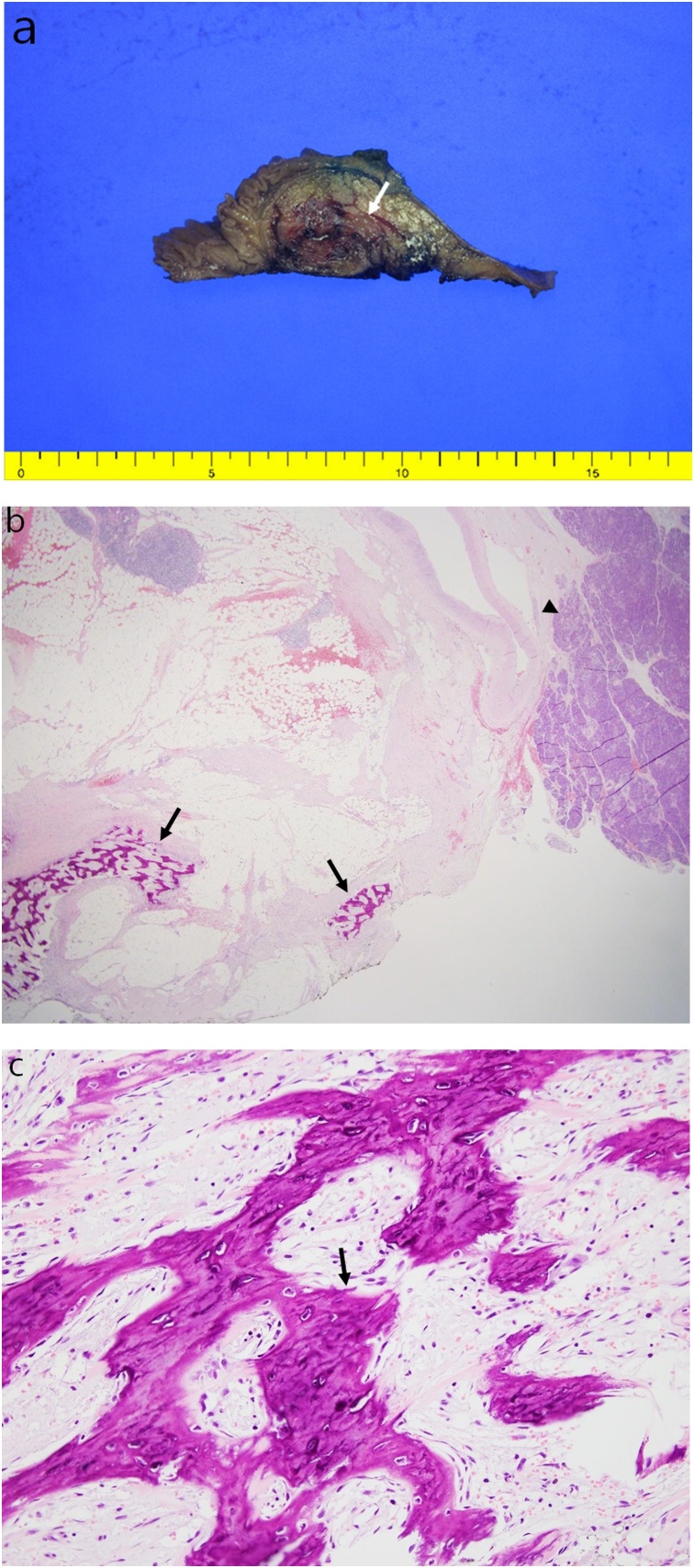
Postoperative histopathological findings. (A) On gross examination, an ill-demarcated, dark-red-to-tan, soft, and fleshy lesion is identified in the extracted peripancreatic soft tissue (arrow). (B) On microscopic examination, calcified lesions (arrow) admixed with fibrous and adipose tissue can be observed within the peripancreatic soft tissues, adjacent to the pancreas (arrowhead). (Hematoxylin and Eosin, 12.5×). (C) Metaplastic bone deposits (arrow) without cellular atypia (Hematoxylin and Eosin, 200×).

The histopathology report confirmed the diagnosis of heterotopic ossification with extensive fibrosis of peripancreatic soft tissue. Grossly, we identified an ill-demarcated, dark-red-to-tan, soft, and fleshy lesion in the periampullary region (Fig. 2A). Microscopic examination revealed calcified lesions admixed with fibrous and adipose tissue, within the peripancreatic soft tissue. However, the metaplastic bone deposits did not show any atypia (Fig. 2B,C). The postoperative course was uneventful, and the patient was discharged 2 weeks after the operation.

## 3 Discussion

This report highlights the case of a patient with spontaneous HMO, which is an extremely rare disease presentation that can involve the periampullary region and have diagnostic features that mimic those of pancreatic cancer.

Heterotopic ossification (HO) is defined as the formation of bone in non-skeletal tissues and classified into 2 subgroups- hereditary and nonhereditary [1]. Nonhereditary HO (NHHO) is usually associated with trauma, tissue injuries, infection, or surgery [1,5,6]. In 1989, Mirra defined the occurrence of HO in soft tissues as “myositis ossificans” [7]. Intra-abdominal HO was first described by Wilson et al. in 1999 [2]. Since then, there have been a few reports of HMO, but most affected patients had a history of trauma, infection, or prior surgery. Only 7 cases of spontaneous HMO have been described in the literature to date (Table 1) [2,[8], [9], [10], [11]]. Although there are various theories regarding the pathophysiological mechanisms of heterotopic bone formation in NHHO, the etiopathogenesis of spontaneous HMO remains unclear [1,12].

**Table 1:** Summary of reported cases of spontaneous heterotopic mesenteric ossification in existing literature.

Reference (year)	Age (years)	Sex	Trauma/operation	Clinical presentation	Diagnostic procedure
Wilson et al. [2] (1999)	43	Male	None	Small bowel obstruction	Surgery
	80	Male	None	Cholelithiasis	Surgery
Comperat et al. [8] (2004)	64	Male	None	Small bowel obstruction	Surgery
	76	Female	None	Abdominal mass	Surgery
Bosker et al. [9] (2004)	70	Male	None	Right flank pain	Surgery
Bovo et al. [10] (2004)	76	Male	None	Small bowel obstruction	Surgery
Deryk et al. [11] (2008)	69	Male	None	Abdominal mass	Needle biopsy
Present case (2020)	60	Male	None	Duodenal stenosis	Surgery

It is difficult to diagnose HMO preoperatively. However, detecting trabecular architecture and dystrophic calcifications on a CT scan may provide confirmatory evidence of ossification [9,13]. While 2 of the reported cases involved patients, who were incidentally diagnosed with high-density lesions on CT scans, we found no suspiciously ossified lesions in the imaging studies in this case. HMO patients are known to present with symptoms of small bowel obstruction. While 5 of the previously reported cases involved patients presenting with abdominal symptoms due to small bowel obstruction, our patient experienced recurrent nausea and vomiting caused by the stenosis of the third part of the duodenum. The duodenal stenosis was associated with severe fibrosis, forming a mass-like lesion around the uncinate process of the pancreas. Thus, the appearance of the lesion mimicked that of pancreatic head cancer, a diagnosis suggested by results of both EGD and CT. However, histopathological examination revealed severe inflammation and HO of the peripancreatic region.

In HMO, surgical management is recommended, and recurrence is rare. As there are a few reports on the effectiveness of additional therapy for prevention of recurrence after surgery, further research is indicated in this regard [1,3,12].

## 4 Conclusions

Spontaneous HMO involving peripancreatic tissue is rare disease. The peripancreatic HMO with severe fibrosis can occur duodenal stenosis, and it is mimicking periampullary cancer. However, the preoperative diagnosis of spontaneous HMO is difficult, and a diagnosis confirmed after surgery.

## Conflicts of interest

The authors of this work have nothing to disclose.

## Sources of funding

This research did not receive any specific grant from funding agencies in the public, commercial, or not-for-profit sectors.

## Ethical approval

This is a case report study and ethical approval not required.

## Consent

Written informed consent was obtained from the patient for publication of this case report and accompanying images. A copy of the written consent is available for review by the Editor of this journal.

## Author contribution

Conceptualization: Hanlim Choi, Dong Hee Ryu.

Data curation: Hanlim Choi, Chang Gok Woo, Ki Bae Kim.

Investigation: Dong Hee Ryu.

Supervision: Dong Hee Ryu, Jae-Woon Choi.

Writing – original draft: Hanlim Choi.

Writing – review & editing: Jae-Woon Choi, Dong Hee Ryu, Chang Gok Woo.

## Registration of research studies

Not applicable.

## Guarantor

The guarantor is Jae-Woon Choi.

## Provenance and peer review

Not commissioned, externally peer-reviewed.
